# Selective deletion of SHIP-1 in hematopoietic cells in mice leads to severe lung inflammation involving ILC2 cells

**DOI:** 10.1038/s41598-021-88677-8

**Published:** 2021-04-28

**Authors:** Xujun Ye, Fengrui Zhang, Li Zhou, Yadong Wei, Li Zhang, Lihui Wang, Haiying Tang, Zi Chen, William G. Kerr, Tao Zheng, Zhou Zhu

**Affiliations:** 1 Section of Allergy and Clinical Immunology, Yale University School of Medicine, 333 Cedar Street, TAC S469C, New Haven, CT 06510 USA; 2 Department of Internal Medicine, Zhongnan Hospital of Wuhan University, Wuhan, 430071 China; 3 Department of Microbiology and Immunology, SUNY Upstate Medical University, Syracuse, NY 31210 USA; 4 Department of Molecular Microbiology and Immunology, Department of Pediatrics, Brown University Medical School, Providence, RI 02918 USA

**Keywords:** Asthma, Inflammation, Lymphocytes, Immunology, Innate immune cells, Innate lymphoid cells

## Abstract

Src homology 2 domain–containing inositol 5-phosphatase 1 (SHIP-1) regulates the intracellular levels of phosphotidylinositol-3, 4, 5-trisphosphate, a phosphoinositide 3–kinase (PI3K) product. Emerging evidence suggests that the PI3K pathway is involved in allergic inflammation in the lung. Germline or induced whole-body deletion of SHIP-1 in mice led to spontaneous type 2-dominated pulmonary inflammation, demonstrating that SHIP-1 is essential for lung homeostasis. However, the mechanisms by which SHIP-1 regulates lung inflammation and the responsible cell types are still unclear. Deletion of SHIP-1 selectively in B cells, T cells, dendritic cells (DC) or macrophages did not lead to spontaneous allergic inflammation in mice, suggesting that innate immune cells, particularly group 2 innate lymphoid cells (ILC2 cells) may play an important role in this process. We tested this idea using mice with deletion of SHIP-1 in the hematopoietic cell lineage and examined the changes in ILC2 cells. Conditional deletion of SHIP-1 in hematopoietic cells in Tek-Cre/SHIP-1 mice resulted in spontaneous pulmonary inflammation with features of type 2 immune responses and airway remodeling like those seen in mice with global deletion of SHIP-1. Furthermore, when compared to wild-type control mice, Tek-Cre/SHIP-1 mice displayed a significant increase in the number of IL-5/IL-13 producing ILC2 cells in the lung at baseline and after stimulation by allergen Papain. These findings provide some hints that PI3K signaling may play a role in ILC2 cell development at baseline and in response to allergen stimulation. SHIP-1 is required for maintaining lung homeostasis potentially by restraining ILC2 cells and type 2 inflammation.

## Introduction

Allergic asthma is a chronic inflammatory disorder of the airways characterized by type 2 immunity-biased inflammatory responses. Increased levels of Th2 cytokines IL-4, IL-5, and IL-13 are underlying molecular basis of the pathological features, including eosinophilic infiltration, mucus hyperproduction, antibody class-switching to IgE, and airway remodeling^1^.

Src homology 2 domain–containing inositol 5-phosphatase 1 (SHIP-1) is one of the phosphatases that control the intracellular levels of the phosphoinositide 3–kinase (PI3K) product phosphotidylinositol-3, 4, 5-trisphosphate. SHIP-1 functions as a negative regulator of cytokine and immune receptor signaling^2–7^. Emerging evidence suggests that the PI3K signaling pathway may be involved in the pathogenesis of allergic inflammation in the lung^8–14^. Germline and inducible global knockout (KO) of SHIP-1 in mice caused spontaneous pulmonary inflammation and enhanced sensitivity to allergic inflammation^15–20^. However, studies using conditional knockout mice to selectively delete SHIP-1 in B cells, T cells, dendritic cells (DC), or myeloid cells did not lead to spontaneous allergic inflammation in the lung or other tissues^21–24^. So far, the cell types and the mechanisms through which SHIP-1 regulates lung inflammation are still unclear.

Group 2 innate lymphoid cells or ILC2 cells (previously termed natural helper cells, nuocytes, or Ih2 cells)^25^, recently discovered in the gut and airway mucosa of mice and man, are rapid and potent producers of type 2 inflammatory cytokines IL-5 and IL-13^26–32^. Treatment of mice with a small molecule inhibitor of SHIP-1 induces super-normal production of these two cytokines^33^, suggesting that SHIP-1 is associated with type 2 inflammation. Recently, a study found that leptin may promote ILC2 cells through activation of the PI3K signaling^34^. Thus, we hypothesized that SHIP-1 regulates ILC2s in the process of allergic inflammation. To explore this possibility, we selectively deleted SHIP-1 in the hematopoietic stem cells and examined the effects on the development of allergic airway inflammation and whether the number of ILC2 cells in the lung tissues were altered at basal level and after allergen challenge. Our data revealed that mice lacking SHIP-1 in hematopoietic stem cells developed spontaneous allergic inflammation in the lung with increased ILC2s, suggesting that SHIP-1 plays an important role in regulating ILC2s in the generation of allergic airway inflammation.

## Materials and methods

### Animals

Wild type (WT), Tek-Cre (also called Tie2-Cre, Stock number 008863), and VEC-Cre (Stock No. 017968) transgenic mice on C57BL/6 genetic background were purchased from the Jackson Laboratory (Bar Harbor, ME). Tek-Cre mice were initially generated and used to target endothelial cells as described by Kisanuki et al.^35^. However, it was realized that high levels of Tek-Cre recombination could be seen in the hematopoietic progenitor cells and Loxp floxed conditional genes can be selectively deleted by Cre in both endothelial cells and hematopoietic progenitor cells^35–38^. The SHIP-1 floxed mice on C57BL/6 genetic background were generated as previously described^17^. Cross breeding Tek-Cre or VEC-Cre mice with SHIP-1 floxed mice to homozygous resulted in Tek-Cre/SHIP-1 or VEC-Cre/SHIP-1 mice. Mice were used at 3 to 8 weeks of age. All mice were housed in cages with microfilters in a specific pathogen-free environment. All procedures performed on mice were in accordance with the National Institutes of Health guidelines for humane treatment of animals and were approved by the Yale University Institutional Animal Use and Care Committee. This study was carried out in compliance with the ARRIVE guidelines.

### Papain-induced allergic airway inflammation

WT and Tek-Cre/SHIP-1 mice were anaesthetized with ketamine/xylazine (i.p.) and exposed intranasally to 25 μg Papain (Calbiochem, San Diego, CA) in 20 μL PBS on day 0 and day 7 as described by Wilhelm et al., for induction of innate immune responses^39^. Twenty-four hours after last Papain challenge (day 8), mice were sacrificed, and lung tissues and cells were collected for evaluation.

### Lung tissues and bronchoalveolar lavage fluid samples

Lung tissue and bronchoalveolar lavage (BAL) fluid samples were obtained as previously described^40,41^. Briefly, mice were anesthetized, the trachea was isolated by blunt dissection, and a small-caliber tubing was inserted and secured in the airway. Four successive volumes of 0.5 mL of PBS were instilled and gently aspirated and pooled. BAL fluid samples were centrifuged, and supernatants were stored at − 80 °C until use. For histology and pathology, the lungs were perfused with cold PBS through the right ventricle with cut vena cava until the pulmonary vasculature was cleared of blood and the lung was inflated with fixatives. For RNA and protein analyses the whole lung was excised. BAL cells were enumerated and classified after cytospin and Diff-Quik staining (Dade Behring, Deerfield, IL). Cytokines and chemokines in the BAL fluid and lung tissue samples were determined by RT-PCR, real-time PCR, and ELISA.

### Histological and pathological evaluation

Lungs and spleens were collected and fixed in 10% buffered formalin and paraffin embedded. Tissue sections of 5-μm thick were stained with hematoxylin and eosin (H&E), Alcian blue, Prussian blue, Masson’s trichrome for histological and pathological evaluations^41^. Sample slides from WT, Tek-Cre/SHIP-1, and VEC-Cre/SHIP-1 mice under comparison were evaluated by a pathologist in a blinded fashion and further confirmed by the analysis of the optical density of the images as outlined and quantified by application of the NIH ImageJ software^42^ and its associated color deconvolution plugin^43,44^. Together, a grading system of pathological changes in the lung was used similar to that described previously^45,46^. Briefly, scores for lung inflammation (H&E), mucous metaplasia (Alcian blue), lung fibrosis (Trichrome), and hemosiderin (Prussian blue) were determined: “−” or Negative, as no inflammatory cell infiltration ; “+” or Low Positive, as inflammatory cell infiltration involvement of < 25% of the lung; “++” or Positive, as between 25 and 50%; “+++” or High Positive, as involvement of > 50% of the lung. Similarly, for lung fibrosis, trichrome positive cells and areas were measured. For hemosiderin deposition, Prussian blue positive cells were determined. For mucous hyperplasia/metaplasia, Alcian blue positive cells in the airway epithelium were determined.

### RNA isolation and mRNA analysis

Total cellular RNA from lungs was extracted using Trizol Reagent (Invitrogen, Carlsbad, CA) with a TissueLyser II (Qiagen, Valencia, CA). The mRNA of specific genes, CCL2 (MCP-1), CCL11 (Eotaxin-1), Arginase-1, Angiopoietin-1, IL-5 and IL-13, was evaluated by RT-PCR and quantitative RT-PCR with specific primers^47^. For RT-PCR, amplified PCR products were analyzed by electrophoresis, and the intensity of the bands and the ratio of specific mRNA to β-Actin were analyzed with the Bio-Rad Gel Doc system and the Quantity One 4.4.1 software (Bio-Rad Laboratories, Hercules, CA). The ∆∆Ct method was used for quantitative PCR. Reactions were carried out in an ABI 7900 real-time PCR system (Life Technologies, Carlsbad, CA) and values were expressed relative to house-keeping gene GAPDH. Primer sequences for RT-PCR and quantitative real-time PCR are shown in Table 1.

**Table 1:** Primers for real-time PCR and RT-PCR.

Gene	Forward	Reverse	Product (bp)
**Real-Time PCR**			
IL-5	AGATTCCCATGAGCACAGTG	TGTCTAGCCCCTGAAAGATTTC	150
IL-13	GCATGGTATGGAGTGTGGAC	ATTGGAGATGTTGGTCAGGG	74
CCL2	GTCCCTGTCATGCTTCTGG	GCTCTCCAGCCTACTCATTG	144
Arginase 1	AAGAATGGAAGAGTCAGTGTGG	GGGAGTGTTGATGTCAGTGTG	132
Angiopoietin-1	GAGGATTGAGCTGATGGACTG	ACCGTGTAAGATCAAGCTGC	148
GAPDH	ACAACTTTGGCATTGTGGAA	GATGCAGGGATGATGTTCTG	133
**RT-PCR**			
CCL 11	CTCCACAGCGCTTCTATTCC	CTTCTTCTTGGGGTCAGCAC	228
CCL2	AGGTCCCTGTCATGCTTCTG	TCTGGACCCATTCCTTCTTG	249
Arginase 1	AACACTCCCCTGACAACCAG	CCAGCAGGTAGCTGAAGGTC	274
IFN-y	ATCTGGAGGAACTGGCAAAA	TGAGCTCATTGAATGCTTGG	247
IL-33	GCTGCGTCTGTTGACACATT	GACTTGCAGGACAGGGAGAC	204
Angiopoietin-1	AGGCTTGGTTTCTCGTCAGA	CCTTTTTGGGTTCIGGCATA	278
GAPDH	AACTTTGGCATTGTGGAAGG	ACACATTGGGGGTAGGAACA	224

### Cytokines and chemokines

The levels of cytokines and chemokines (IL-13, CCL2) in the BAL fluid were measured using commercial ELISA kits per the manufacturer’s instructions (R&D Systems, Minneapolis, MN).

### Isolation of mouse lung and spleen tissue cells

Lung tissue cells and leukocytes for flow cytometry analysis were isolated as described previously^48^. Mice were sacrificed and lungs were perfused, minced, and incubated in 5 mL of pre-warmed RPMI 1640 medium containing 0.01% DNase I (Roche) and 650 units per ml collagenase I (Worthington) in a 37 °C shaking incubator for 30 min. The digested lung tissues were passed through a 70-μm strainer and centrifuged at 400* g* for 5 min. The cells were resuspended in 5 mL of 40% Percoll underlaid with 5 mL of 60% Percoll and centrifuged at 800* g* for 20 min. Lung cells were collected from the interface. RBC were lysed in 5 mL of ammonium chloride, centrifuged and the pellets were resuspended in 1 mL PBS containing 2% FBS and viable cells were counted. Similarly, splenocytes were isolated for flow cytometry analysis.

### Flow cytometry analysis

Lung and spleen tissue cells were analyzed using flow cytometry as described previously^24^. Briefly, cell samples were incubated in staining buffer with 10% goat serum and 5 µg/mL anti-CD16/CD32 to block nonspecific binding. Antibodies to IL-13, IFN-γ, FoxP3, CD11c, MHC class II, CD4, CD11b, and CD45R/B220 were purchased from eBioscience (San Diego, CA). Antibodies to CD103, CD40, Siglec-F, CD86, and CD45 were from BD Biosciences (San Jose, CA). Anti-neutrophil antibody (7/4) was purchased from Abcam (Cambridge, MA). SHIP-1 antibody (P1C1) was from Santa Cruz Biotechnology (Dallas, TX) and conjugated to PE or Alexa Fluor 647 (AbLab, Vancouver, British Columbia, Canada). ILC2 cells in lung and spleen were identified as Lineage^−^B220^−^CD127^+^CD25^+^CD90.2^+^T1/ST2^+^ cells, which are primarily Sca-1^+^ and CD117^+^. Intracellular staining was performed as described previously with modifications^24^. Dead cells were excluded using eFluor fixable viability dyes (eBioscience). Samples were acquired on a BD LSR II Flow Cytometer, and data analysis was performed using the FlowJo software (Tree Star, Ashland, OR).

### Intracellular cytokine analysis

Intracellular cytokines were analyzed as described previously^24^. Lung tissues were minced and digested in 200 U/mL collagenase IV (Sigma, St Louis, MO) for 1 h at 37 °C and passed through a 70-µm cell strainer. RBCs were lysed and leukocytes were enriched with Percoll (Sigma) separation. Isolated leukocytes were resuspended in culture medium (Iscove modified Dulbecco medium with 10% FBS, penicillin/streptomycin, and 150 mmol/L monothioglycerol) containing 750 ng/mL ionomycin and 50 ng/mL phorbol 12-myristate 13-acetate (Sigma) in the presence of brefeldin A (eBioscience) for 4 h before intracellular staining for flow cytometry. We used the Intracellular Fixation & Permeabilization Buffer Set or the FoxP3/Transcription Factor Buffer Set from eBioscience.

### Statistical analysis of data

Data were analyzed with GraphPad Prism 5.0 for Mac OS, GraphPad Software, San Diego, California USA, www.graphpad.com. Student’s t test was used to compare between groups. Differences with p < 0.05 were considered statistically significant. Data were expressed as Mean ± SD unless otherwise indicated.

## Results

### Targeted deletion of SHIP-1 in hematopoietic cells

**Figure 1:**
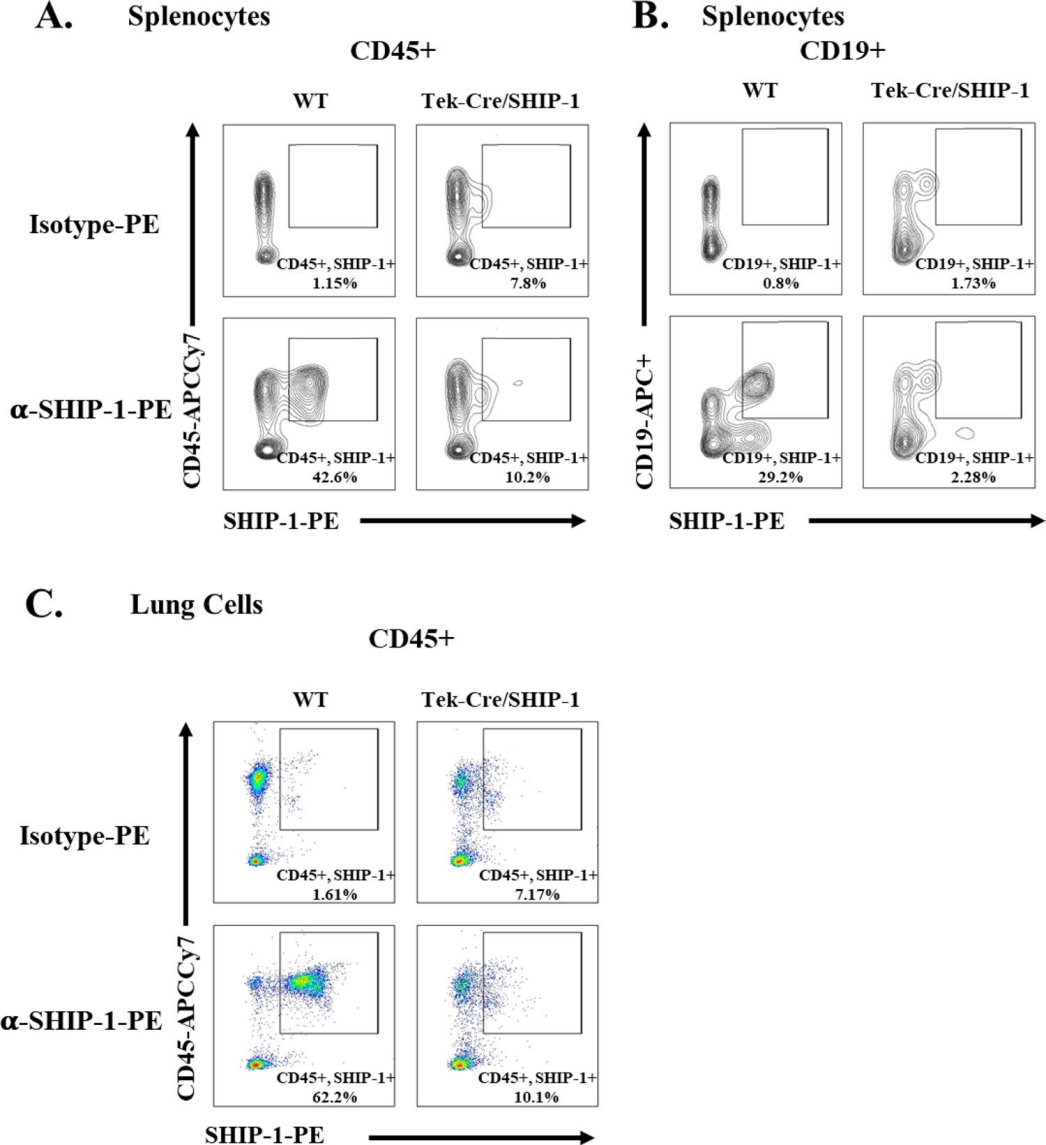
Flow cytometry analysis of SHIP-1 expression in tissue cells. Cells were prepared from spleen and lung tissues of WT and Tek-Cre/SHIP-1 mice 3–8 weeks of age and stained with labeled antibodies to cell markers (CD45 and CD19) and intracellular SHIP-1 and analyzed by flow cytometry. (**A**) Splenocytes from WT and Tek-Cre/SHIP-1 mice stained with anti-CD45 and anti-SHIP-1 antibodies; (**B**) CD45 + gated splenocytes stained with anti-CD19 and anti-SHIP-1; (**C**) Lung tissue cells from WT and Tek-Cre/SHIP-1 mice stained with anti-CD45 and anti-SHIP-1*.* Shown are representative results of 3 separate experiments. Numbers are percentages of cells.

The Tek (Tie2) gene is consistently expressed in endothelial cells. But it is also transiently expressed in other cell types, such as hematopoietic cells. The Tek-Cre mice were originally generated to target genes in endothelial cells. It was realized later that hematopoietic progenitor cells are also affected^35–37^. Cross breeding Tek-Cre mice to SHIP-1 floxed (SHIP-1 f/f) mice generated Tek-Cre/SHIP-1 f/f or Tek-Cre/SHIP-1 mice. We examined the SHIP-1 expression in various cells in these mice and compared with WT mice. Spleen and lung cells were prepared and analyzed by flow cytometry with staining for several cell markers and for intracellular SHIP-1 protein. As shown in Fig. 1, in the spleen of WT mice, more than 40% of the cells were CD45 + leukocytes and those cells expressed SHIP-1. However, only about 10% of the cells from the spleen of Tek-Cre/SHIP-1 mice were CD45 + SHIP-1 + , which was close to the background reading in isotype control (7.8%) (Fig. 1A). Similarly, a large proportion (29%) of the splenocytes from WT mice were CD19 + B cells that expressed SHIP-1, whereas only few cells (2%), again at the background level, were CD19 + SHIP-1 + in Tek-Cre/SHIP-1 mice (Fig. 1B). SHIP-1 expression in CD3 + T cells from Tek-Cre/SHIP-1 mice were significantly lower than T cells from WT mice (data not shown). Analysis of the lung cells showed that the majority of the cells (62%) from WT mice were CD45 + SHIP-1 + , but only a small percentage (8%), like isotype control (7%), of the cells from Tek-Cre/SHIP-1 mice were stained CD45 + SHIP-1 + (Fig. 1C). These results demonstrate that the Tek promoter directed Cre recombinase efficiently deleted the SHIP-1 gene in leukocytes.

### SHIP-1 deletion in leukocytes led to severe lung and airway inflammation

**Figure 2:**
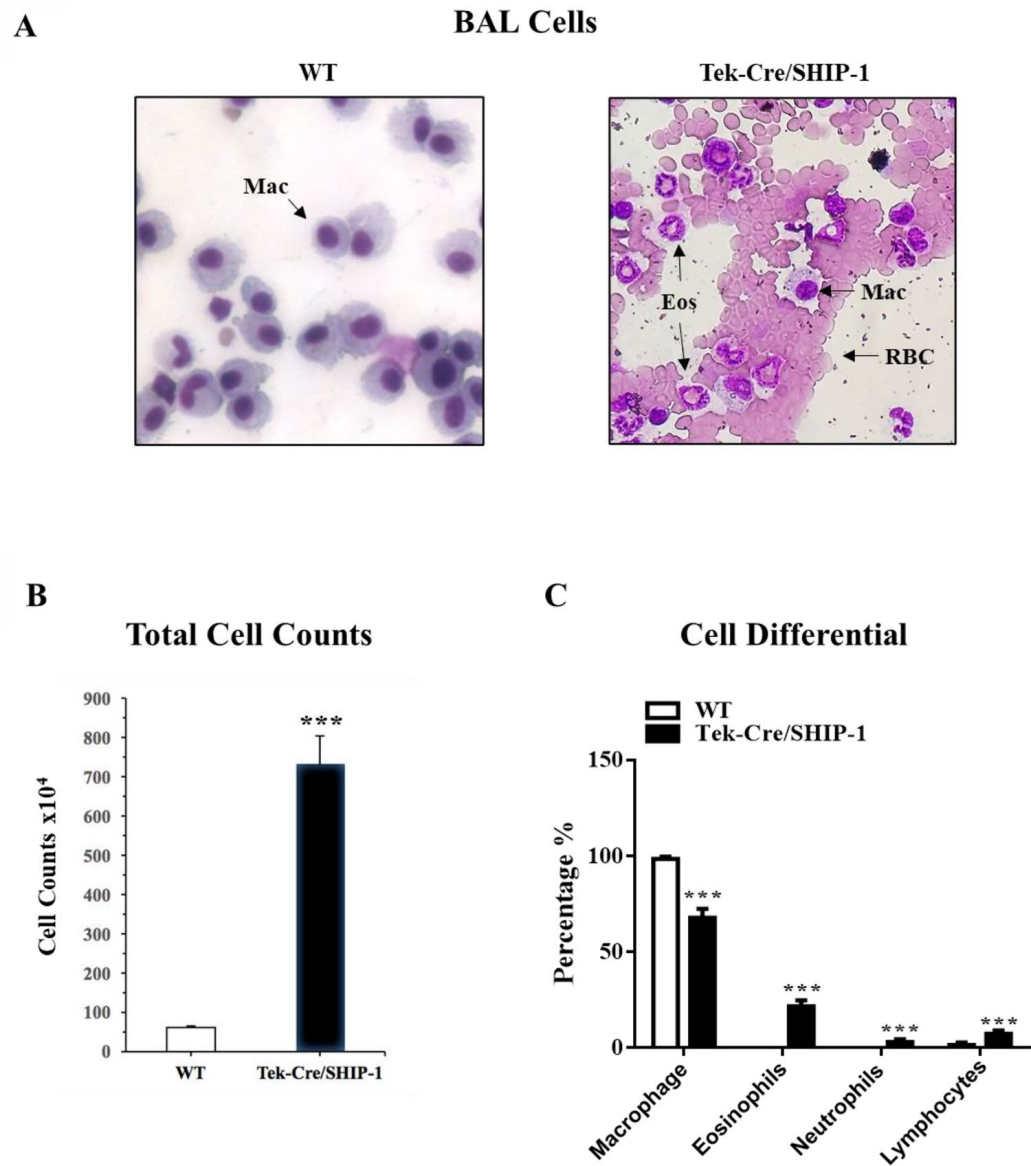
Analysis of bronchoalveolar lavage (BAL) cells. (**A**) Diff-Quik stained BAL cells (WT and Tek-Cre/SHIP-1 mice). (**B**) BAL total cell counts and (**C**) Cell differential were obtained from WT mice and Tek-Cre/SHIP-1 mice. Labels: *Mac* alveolar macrophages; *Eos* eosinophils; *RBC* red blood cells. Open bar: WT mice; Filled bar: Tek-Cre/SHIP-1 mice. (n = 5–6 mice for each group, ***P < 0.001 vs WT group).

**Figure 3:**
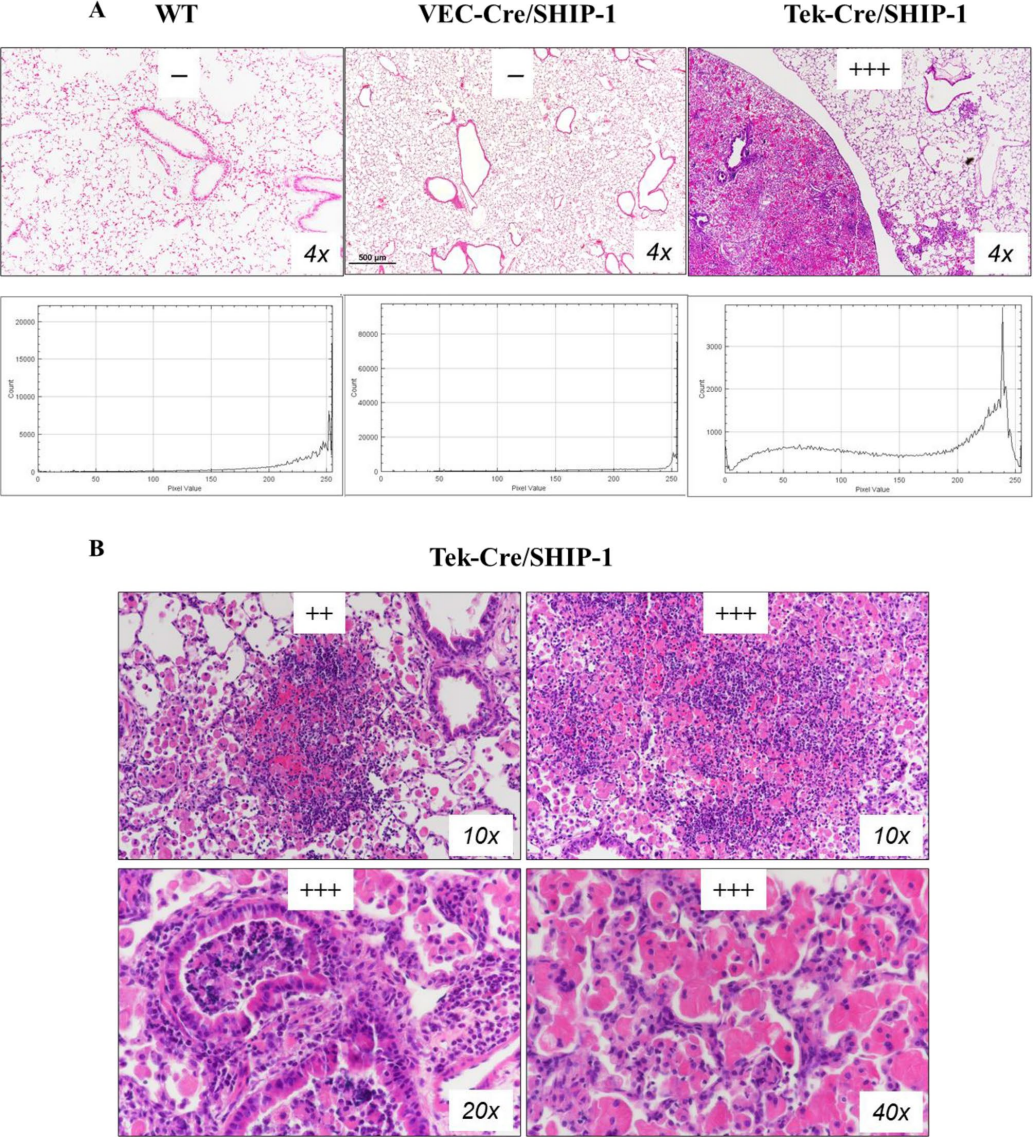
Pulmonary histopathology of Tek-Cre/SHIP-1 mice. Shown are Hematoxylin and eosin (H&E) stained lung sections from (**A**) WT, VEC-Cre/SHIP-1, and Tek-Cre/SHIP-1 mice and (**B**) Tek-Cre/SHIP-1 mice. Lung sections from WT and VEC-Cre/SHIP-1 mice showed normal structure of airways and alveoli. In contrast, mild, medium, and severe cellular infiltration was seen in the airways and lung parenchyma of Tek-Cre/SHIP-1 mice. All Tek-Cre/SHIP-1 mice examined displayed inflammatory lung pathology. Pathology scores were assigned by a pathologist and through application of the NIH ImageJ software and its associated color deconvolution plugin.

After birth, Tek-Cre/SHIP-1 mice appeared to be smaller in size in the litter. By the time of weaning (3–4 weeks of age), Tek-Cre/SHIP-1 mice were runted and showed labored breathing. By the age of 3 months, all Tek-Cre/SHIP-1 mice became sick. VEC-Cre/SHIP-1 mice did not appear to be abnormal. We performed bronchoalveolar lavage (BAL) on WT, VEC-Cre/SHIP-1 and Tek-Cre/SHIP-1 mice and obtained lung tissues for analyses. BAL fluid samples from WT mice and VEC-Cre/SHIP-1 showed macrophages without eosinophils or red blood cells. However, BAL samples from Tek-Cre/SHIP-1 mice showed large numbers of red blood cells and inflammatory cells, including macrophages, eosinophils, and neutrophils (Fig. 2A–C). Histology and pathology evaluation of the lung tissues showed that lungs from WT and VEC-Cre/SHIP-1 mice were normal and no spontaneous abnormalities in other organs or tissues. Therefore, we analyzed the VEC-Cre/SHIP-1 mice in a separate study. We found that the VEC-Cre/SHIP-1 mice with endothelial deletion of SHIP-1 had significantly increased fibrotic responses to bleomycin challenge^49^. However, in this study, we found that all Tek-Cre/SHIP-1 mice developed spontaneous lung inflammation. In and around the airways and in the lung parenchyma, small clusters and consolidated areas with inflammatory cells can be seen. Some infiltration was massive, involving the whole lung lobes (Fig. 3). The most prominent cells were macrophages. Other cells included eosinophils, neutrophils, and lymphocytes. Histopathological analyses by a pathologist in a blinded fashion and by using the NIH ImageJ software found that Tek-Cre/SHIP-1 mice developed positive (++) to high positive (+++) inflammatory infiltrates in the lung (Fig. 3). The pulmonary pathology, a type 2-like inflammatory response, is very similar to what has been described previously by us and others in the germline and induced global SHIP-1 knockout mice^16,19,50^. Interestingly, this observation is different from the reported lack of a spontaneous phenotype seen in B cell, T cell, DC, and myeloid cell-specific SHIP-1 knockout mice^21–24^.

### Mucous hyperplasia/metaplasia appeared in Tek-Cre/SHIP-1 mice

**Figure 4:**
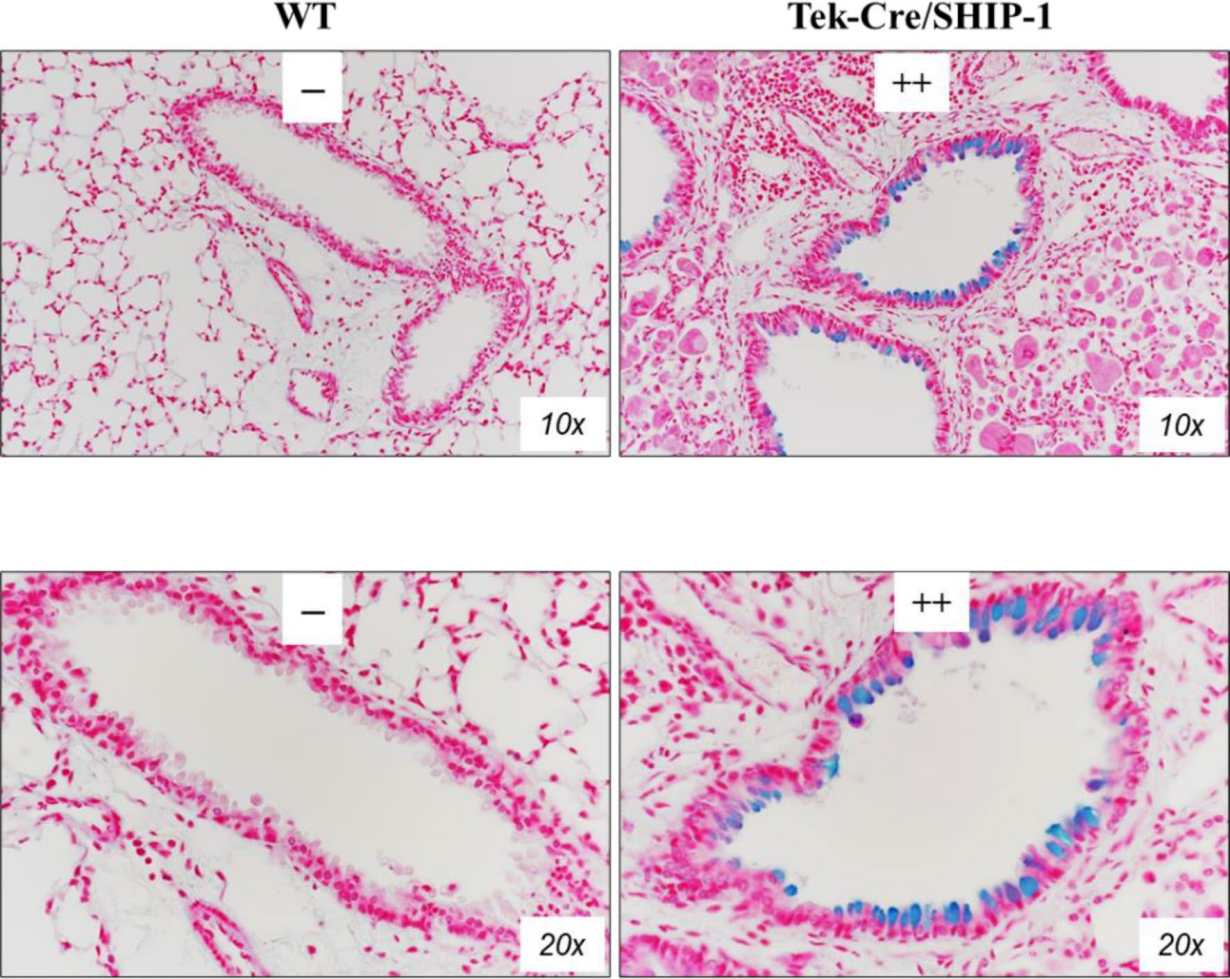
Mucous hyperplasia/metaplasia. Alcian Blue staining of lung sections from WT and Tek-Cre/SHIP-1 mice are shown. Alcian Blue-positive cells in the airways were readily seen in the lung of Tek-Cre/SHIP-1 mice (n = 4 in each group). Pathology scores were assigned by a pathologist.

To determine whether mucous hyperplasia was present, we analyzed lung sections from WT and Tek-Cre/SHIP-1 mice using Alcian blue staining for mucin. As expected, no Alcian blue-positive staining was found in the lung sections of WT mice. In contrast, markedly increased Alcian blue-positive cells were readily seen in the airways of Tek-Cre/SHIP-1 mice, indicating mucous hyperplasia/metaplasia (Fig. 4).

### SHIP-1 deletion induced lung fibrosis

**Figure 5:**
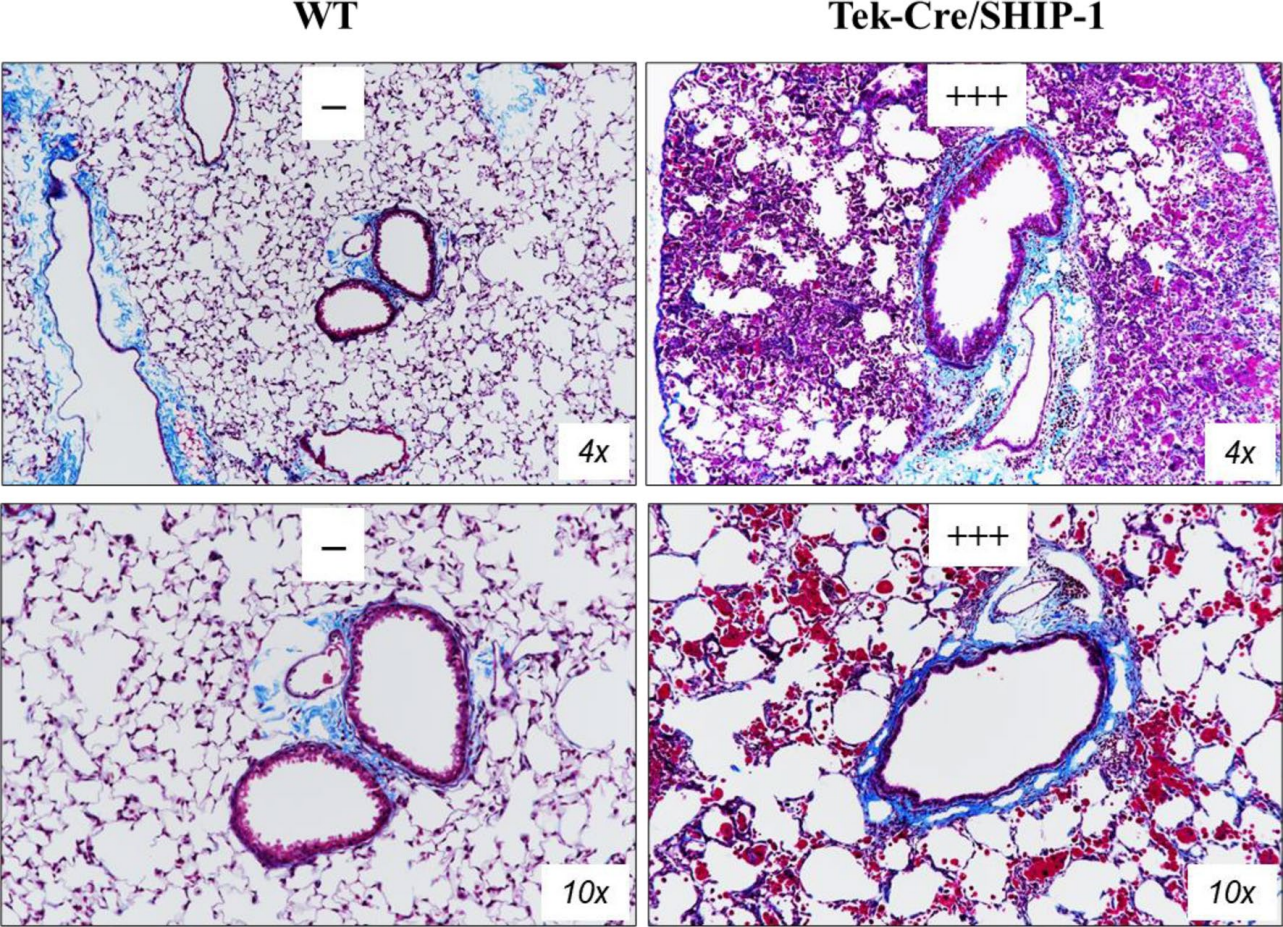
Pulmonary fibrosis in Tek-Cre/SHIP-1 mice. Masson’s trichrome staining of lung sections from WT and Tek-Cre/SHIP-1 mice are shown (n = 4 in each group). Pathology scores were assigned by a pathologist and through application of the ImageJ software and its associated color deconvolution plugin.

To assess whether there were fibrotic changes, Masson’s trichrome staining was used to stain the lung sections from WT and Tek-Cre/SHIP-1 mice. Trichrome stained thin layers of collagen around the airways could be seen in the lung tissues of WT mice, which is normal (Fig. 5). However, markedly increased collagen deposition was seen in the subepithelial areas of the airways and in the parenchyma of the Tek-Cre/SHIP-1 mouse lung (Fig. 5). Pathological analysis and software assisted analysis by ImageJ confirmed the severity of the lung fibrosis in Tek-Cre/SHIP-1 mice.

### SHIP-1 deletion caused chronic pulmonary hemorrhage

**Figure 6:**
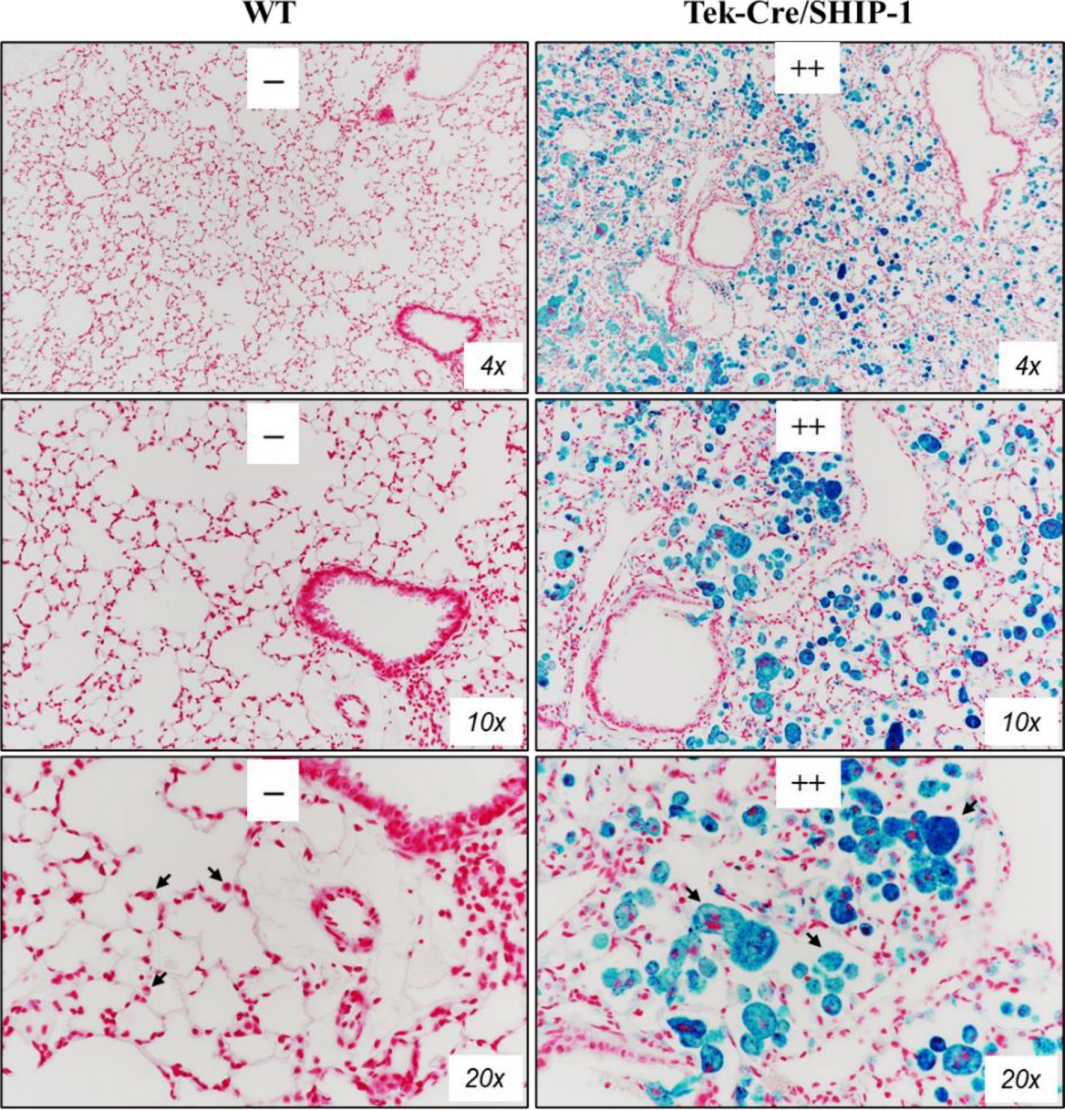
Hemosiderin deposition in the lung tissues of Tek-Cre/SHIP-1 mice. Prussian blue stained lung sections from WT and Tek-Cre/SHIP-1 mice are shown under different magnifications. Arrows point to macrophages in WT lung and groups of macrophages in Tek-Cre/SHIP-1 lung. Pathology scores were assigned by a pathologist and through application of the ImageJ software and its associated color deconvolution plugin.

We noticed that red blood cells (RBC) were readily seen in the BAL samples (Fig. 2A) and in lung tissue sections from Tek-Cre/SHIP-1 mice, in contrast to the BAL samples or lung sections from WT mice. To determine whether this was spontaneous internal pulmonary hemorrhage, we analyzed lung sections from Tek-Cre/SHIP-1 and WT mice using Prussian blue staining for hemosiderin, iron-storage complex formed in macrophages after phagocytosis of RBCs in tissues. As expected, no Prussian blue-positive stained cells were seen in the lung of WT mice. In contrast, markedly increased numbers of Prussian blue stained macrophages were found in the lung parenchyma of Tek-Cre/SHIP-1 mice, indicating severe chronic pulmonary hemorrhage in these mice (Fig. 6). The degrees in positive staining were assessed by pathology analysis and ImageJ software.

### Alterations in pro-inflammatory cytokines and chemokines in the lung

**Figure 7:**
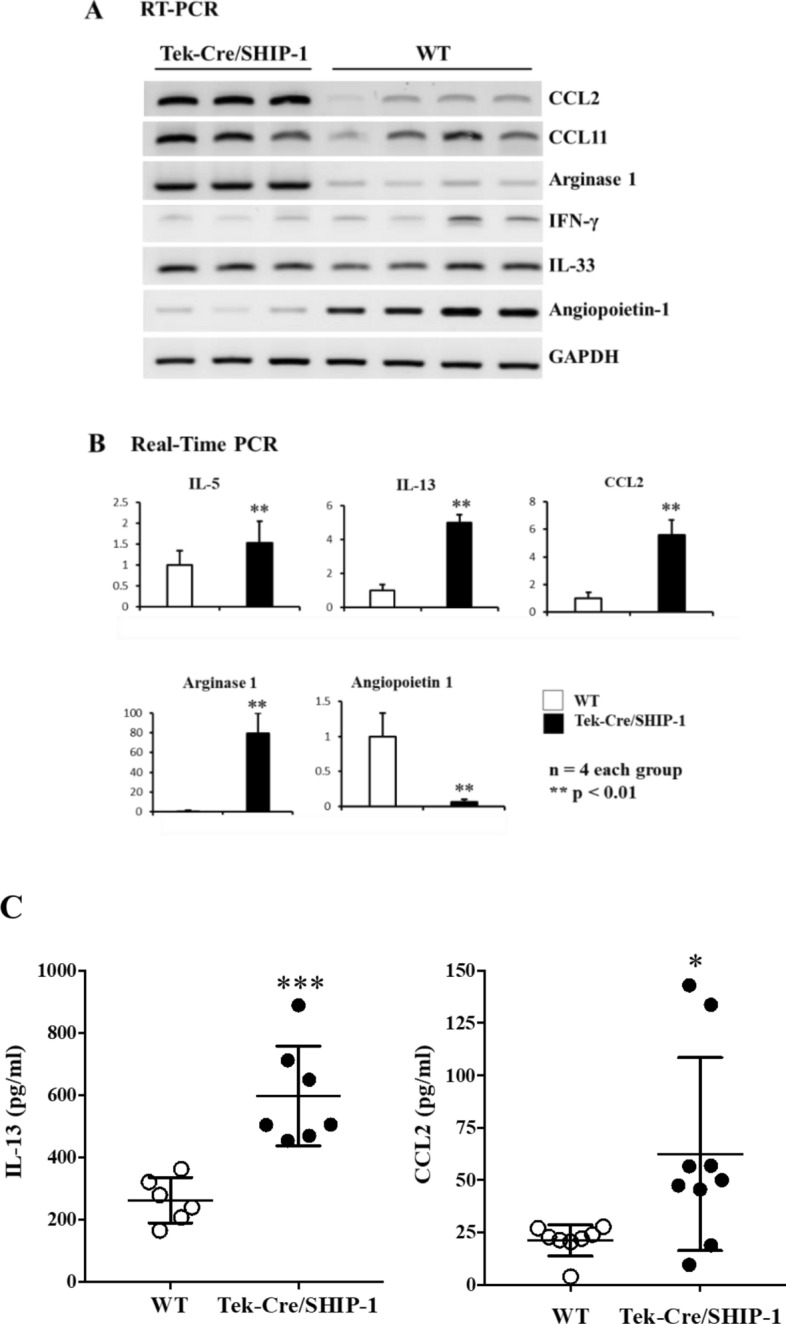
Altered expression of cytokines, chemokines, and factors in the lung. (**A**) RT-PCR analysis of total lung RNA from WT and Tek-Cre/SHIP-1 mice using gene-specific primers is shown. Amplified products were analyzed by electrophoresis. (n = 3–4) (**B**) Real-time quantitative PCR analysis of cytokine and chemokine expression using the double delta method (ΔΔC) (n = 4 for each group) (**p < *0.01* vs. WT*)*. (C) ELISA measurement of IL-13 and CCL2 proteins in the BAL fluid samples (n = 6–9) (*p < 0.05, ***p < *0.001* vs. WT*)*.

Next, we investigated the expression of pro-inflammatory cytokines and chemokines in the lung. As determined by RT-PCR and real-time quantitative PCR (qPCR), compared to those in the WT lung tissues, the expression of CCL2, CCL11, Arginase-1, IL-5, and IL-13 was significantly increased, the expression of IFN-γ and IL-33 did not change, whereas the expression of Angiopoietin-1 was significantly decreased in the lungs of Tek-Cre/SHIP-1 mice (Fig. 7A,B). Measured by ELISA, IL-13 and CCL2 proteins were significantly increased in the BAL fluid of Tek-Cre/SHIP-1 mice compared to WT mice (Fig. 7C). These results showed that changes in the cytokine and chemokine profiles were consistent with observed type 2 inflammatory phenotype in the lung.

### SHIP-1 deletion increased innate lymphoid cells type 2 (ILC2s) in the lung

**Figure 8:**
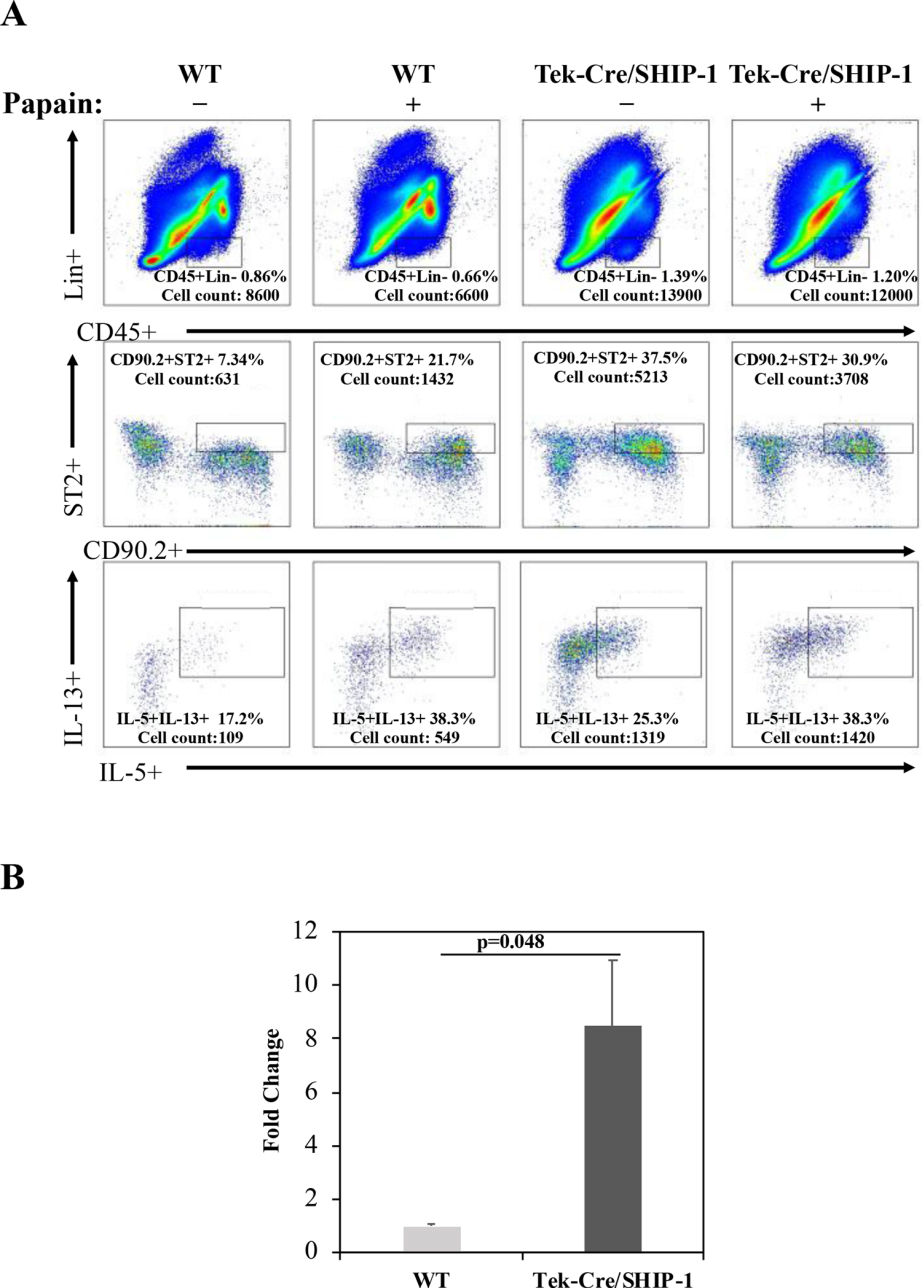
Increased ILC2 cells in the lung tissues of Tek-Cre/SHIP-1 mice. (**A**) Lung tissue samples of WT and Tek-Cre/SHIP-1 mice were analyzed by flow cytometry for ILC2 cells. CD45 + Lineage-ST2 + and IL-5/IL-13-producing ILC2 cells in the lung tissues of WT and Tek-Cre/SHIP-1 mice were analyzed at baseline or after Papain stimulation for 7 days. The percentage and total cell count for each sample are shown. Representative data from 3 independent experiments are shown. (**B**) Comparison of ILC2 cells in the lung of WT and Tek-Cre/SHIP-1 mice at baseline. Shown is fold change in mice (n = 3 each group, *p* = *0.048* using Student’s t test).

It has been reported that B cell, T cell, DC, and myeloid cell-specific deletion of SHIP-1 did not cause spontaneous type 2 like lung inflammation, our attention turned to innate immune cells, specifically ILC2 cells. To determine whether ILC2 cells were involved in the spontaneous pulmonary phenotype in Tek-Cre/SHIP-1 mice, we assessed the changes in ILC2 cells in the lung tissues at baseline and after allergen challenge (Papain). WT and Tek-Cre/SHIP-1 mice were challenged with PBS control or Papain (i.t., 2x) on day 0 and day 7, a short protocol to induce innate immune response. Lung tissues were collected and analyzed on day 8. As shown in Fig. 8, at baseline (PBS), a small population of Lin-ST2 + ILC2s could be seen in the lung tissues of WT mice and among these, a few cells appeared to be IL-5 + and IL-13 + . After Papain challenge, both total ILC2s and IL-5/IL-13-producing ILC2s were significantly increased. However, compared to WT mice, the number of Lin-ST2 + ILC2s and the percentage of cells producing IL-5/IL-13 in the lung of Tek-Cre/SHIP-1 mice were significantly higher at the baseline (~ 7.4-fold higher than those in WT mice) and those have not increased significantly further after Papain stimulation. These results indicate that increased ILC2 cells, particularly IL-5/IL-13-producing ILC2 cells in the lung tissues of Tek-Cre/SHIP-1 mice are probably an important source for the Th2 cytokines and are likely the cell types responsible for the generation of the spontaneous type 2 inflammatory phenotype in these mice.

## Discussion

Studies using mouse strains deficient in PI3K isoforms or specific antagonists demonstrated that the PI3K signaling pathway plays an important role in different cell types in allergen induced allergic inflammatory responses^10,12,14,51–53^. Interestingly, PI3K signaling may play a role in ILC2 cell activity^34^. SHIP-1, as one of the key negative regulators of the PI3K signaling pathway, hydrolyzes the second messenger PI-(3,4,5)-trisphosphate (PIP3), reducing or terminating the downstream signaling. The importance of SHIP-1 in regulating immunological functions has been demonstrated in mice with germline or induced whole-body deletion of SHIP-1^3,16,50,54,55^. Particularly, mice lacking this enzyme develop spontaneous severe eosinophilic crystalline pneumonia and Crohn’s like inflammatory bowel disease with the former characterized as a Th2-like airway inflammation, which is possibly the main cause of shortened lifespan for these mice^16,19,50^. Findings from adoptive transfer experiments suggested that SHIP-1 deficient hematopoietic cells are likely responsible for initiation of the spontaneous pulmonary inflammation, although tissue structural cells may also be involved^55^. To date, however, the cell types that initiated the pulmonary phenotype due to cell-intrinsic SHIP-1 deficiency have not been identified. Several attempts have been made. Studies using conditional knockout mice found that cell-specific deletion of SHIP-1 in macrophages, dendritic cells (DC), B cells and T cells resulted in some changes in cellular and immunological functions, without causing spontaneous lung inflammation^21,23,24,56^. These results suggested that other cell types may be important in the initiation of lung inflammation in SHIP-1 deficient mice. Also, the early and spontaneous nature of the inflammatory phenotype suggest an involvement of innate immune cells. However, a role of innate lymphoid cells type 2 (ILC2) cells has not been examined in this context.

To address this issue, we selectively deleted SHIP-1 in hematopoietic progenitor cells using the Tek promoter controlled Cre recombination in homozygous Tek-Cre/SHIP-1 mice and examined the inflammatory pulmonary phenotype. Tek-Cre directed deletion of genes has been utilized to target endothelial cells and hematopoietic cells^35–37^. Flow cytometry analysis of cells isolated from spleen and lung tissues of WT mice showed that the majority of CD45 + leukocytes express SHIP-1. However, when the SHIP-1 gene was targeted by Tek-Cre, only a small percentage of CD45 + cells from Tek-Cre/SHIP-1 mice remain positive for SHIP-1, although the total numbers of CD45 + cells were comparable in these mice and WT mice. Thus, the Tek-Cre system established conditional knockout of the SHIP-1 gene in leukocytes.

It has been reported that Tek-Cre can lead to deletion of target genes in both hematopoietic cells and endothelial cells^35–37^. To confirm the cell-specific effects, we also generated VEC-Cre/SHIP-1 mice in which Cre is expressed and effective in endothelial cells or endothelial derived blood cells^57^. Interestingly, deletion of SHIP-1 in endothelial cells alone did not result in any spontaneous lung inflammation in VEC-Cre/SHIP-1 mice. Further studies showed that these mice had exaggerated fibrotic responses in bleomycin induced mouse model of lung fibrosis^58^. In contrast, Tek-Cre/SHIP-1 mice developed a spontaneous lung phenotype of eosinophilic pneumonia like that observed in the whole-body knockout mice. Detailed analyses showed that all Tek-Cre/SHIP-1 mice had pulmonary pathology, including lung tissue infiltration of macrophages and eosinophils, mucous metaplasia, and lung fibrosis, which are features of type 2 dominated inflammation and airway remodeling^1^. We also noticed many Prussian blue positive hemosiderin containing macrophages in the lung tissues of Tek-Cre-SHIP-1 mice, a sign of chronic hemorrhaging in the lung (Fig. 6). There is a question whether hemorrhage is related to any effects in endothelial cells. Since endothelial deletion of SHIP-1 alone in VEC-Cre-SHIP-1 mice did not show any lung hemorrhage, it is reasoned that chronic bleeding in the lung was related to or initiated by hematopoietic cells in Tek-Cre/SHIP-1 mice. BAL cellularity and cytokine expression data supported the pathological findings in the lung. Notably, Th2 cytokines IL-5 and IL-13 and chemokines CCL2 (MCP-1) and CCL11 (Eotaxin-1) were significantly increased in the lung of Tek-Cre/SHIP-1 mice. Arginase-1, a marker of M2 cells, was significantly up-regulated. On the other hand, angiopoietin-1 was down-regulated, which may contribute to the perturbation of vascular integrity in the lung of these mice.

M2 macrophages, also known as alternatively activated macrophages, contribute to allergic lung inflammation^59^. Both IL-4 and IL-13 are strong inducers of M2 cells. Studies show that ILC2 cells alone or working together with T cells are able to maintain M2 macrophages for lung immunity^60,61^. In our study, the infiltration of large numbers of macrophages in the lung tissues of Tek-Cre/SHIP-1 mice and the presence of increased type 2 cytokines, particularly IL-13, M2 marker arginase-1, and airway remodeling (inflammation, mucous metaplasia, and fibrosis) indicate an M2 macrophage phenotype in these mice. Further characterization should be carried out to determine the biological meaning of this ILC2-IL-13-M2 phenotype.

Few Tek-Cre/SHIP-1 mice lived past the age of 10 weeks. This is in accordance with the results of survival analysis in a previous study, in which 40% of SHIP-1 KO mice died from lung inflammation by the age of 12 weeks^16,19^.

Although Tek-Cre targets both hematopoietic cells and endothelial cells, deletion of SHIP-1 in hematopoietic cells is responsible for the inflammatory lung phenotype in this study. Whether SHIP-1 deficient endothelial cells in Tek-Cre/SHIP-1 mice may enhance vascular leakage as seen in the lung tissues needs to be further investigated.

Type 2 inflammation can be initiated by Th2 cytokine producing cells, which could be T cells of the adaptive immune system or ILC2 cells of the innate immune system. Adaptive type 2 immune responses require exposure to allergens or parasites and coordination between antigen presenting DCs and T cells. However, deletion of SHIP-1 in DCs or T cells did not induce lung inflammation^21,24^, although combined myeloid and T cell KO of SHIP-1 did lead to lung inflammation^54^. In addition, Tek-Cre/SHIP-1 mice, as well as whole-body SHIP-1 knockout mice, developed the lung phenotype without going through discernable adaptive immunological processes. Interestingly, rescue of lung and gut T cell numbers by Caspase 8 inhibition reduces inflammation at both mucosal sites in SHIP-1 KO mice, suggesting the cellular source of inflammation has multiple components^54^. These findings suggest that innate immune cells, particularly ILC2 cells which produce IL-13 and IL-5, may contribute to the generation of the type 2 inflammatory lung phenotype in Tek-Cre/SHIP-1 mice. Indeed, we found that the number of lineage^-^ST2^+^ IL5/IL-13-producing ILC2 cells in the lung of Tek-Cre/SHIP-1 mice was significantly higher than those in WT mice. With a short-term Papain exposure to elicit an innate immune response, further increased IL-5/IL-13-producing ILC2 cells were observed in the lung of Tek-Cre/SHIP-1 mice compared to WT mice. Increased ILC2 cells could be the source of the increased Th2 cytokines seen in the lung of Tek-Cre/SHIP-1 and whole-body SHIP-1 knockout mice^19^. These findings indicate that the PI3K signaling pathway plays an important role in promoting ILC2 cells, whereas SHIP-1 is required to restrain ILC2 cells under basal conditions and in response to innate immune stimulation.

Type 2 cytokine-producing cells also include mast cells and basophils, usually as effector cells in response to antigen–antibody interaction and as an end result of adaptive immunity. SHIP-1 deficient mice had increased mast cells in the lung^19^ and reconstitution of SHIP-1 deficient mast cells to mast cell deficient mice enhanced allergen induced lung inflammation and antigen-IgE interaction induced anaphylaxis without inducing spontaneous lung inflammation^62^. Thus, SHIP-1 deficient mast cells may act as an enhancer instead of an initiator in these processes. The role of basophils as an initiator of lung inflammation is unknown since there is no study to test this possibility using SHIP-1 deficient basophils.

A recent study showed that IL-33 stimulates Th2 and ILC2 cell production of IL-5 and IL-13 through activation of PI3K p110δ isoform and mTOR^63^, providing evidence that the PI3K signaling pathway is involved in both adaptive and innate type 2 immune responses. So far, the exact role of the PI3K pathway in ILC2 cell development has not been elucidated. Our study here demonstrated that uncontrolled PI3K activity in hematopoietic progenitor cells, including those for ILC2 cells, in SHIP-1 deficient mice is likely the driving force for increased ILC2 cells and increased production of type 2 cytokines in the lung.

Our study has some limitations. It is unclear in which phase ILC2 cells were affected by the lack of SHIP-1. Whether it is in the development, proliferation, recruitment, or function (production and secretion of cytokines) phase? Whether the observed effects are cell intrinsic to ILC2 cells or indirectly initiated by other cell types is still a question. For the role of the PI3K signaling pathway in ILC2 cells, direct measurement of the levels and effects of intracellular inositol phosphates on these cells are important considerations. More sophisticated techniques, such as ILC2 cell-specific deletion of SHIP-1 gene, and intervention with specific inhibitors are required to address these questions.

## Conclusions

Our study demonstrated that mice lacking SHIP-1 selectively in hematopoietic cells develop spontaneous type 2 like inflammation in the lung. Increased ILC2 cells are present in the lung tissues of these mice at basal levels and after allergen stimulation. These findings suggest that SHIP-1 plays an important role in maintaining lung homeostasis, probably through regulating ILC2 cells in the lung. Further studies of the role of the PI3K pathway and SHIP-1 regulation in type 2 innate immune responses will provide valuable insights into potential therapeutic targets for controlling allergic inflammation in diseases such as asthma.

## Supplementary Information

Supplementary Information.

## Abbreviations

BAL: Bronchoalveolar lavage
Cre: Cre recombinase
ILC2 cells or ILC2s: Group 2 innate lymphoid cells
PI3K: Phosphoinositide 3-kinase
PIP3: Phosphotidylinositol-3,4,5-trisphosphate
SHIP-1: Src homology 2 domain–containing inositol-5-phosphatase 1
Tek: Endothelial-specific receptor tyrosine kinase
VEC: VE-cadherin
WT: Wild type
