# Supplementary material for: Selective deletion of SHIP-1 in hematopoietic cells in mice leads to severe lung inflammation involving ILC2 cells
Source: Sci Rep. 2021 Apr 28;11:9220. doi: 10.1038/s41598-021-88677-8 (PMC8080607; doi:10.1038/s41598-021-88677-8)
Supplement: Supplementary file 1 — Supplementary Information. [file 41598_2021_88677_MOESM1_ESM.pdf]

Supplemental Materials

Selective Deletion of SHIP-1 in Hematopoietic Cells in Mice Leads to Severe Lung Inflammation Involving ILC2 Cells

Xujun Ye[1,2], Fengrui Zhang[1], Li Zhou[2], Yadong Wei[1], Li Zhang[1,2], Lihui Wang[1,2], Haiying Tang[1], Zi Chen[1], William G. Kerr[3], Tao Zheng[1,4], and Zhou Zhu[1,4,*]

[1]Section of Allergy and Clinical Immunology
Yale University School of Medicine
333 Cedar Street, TAC S469C
New Haven, CT 06510

[2]Department of Internal Medicine
Zhongnan Hospital of Wuhan University
Wuhan, China 430071

[3]Department of Microbiology and Immunology
SUNY Upstate Medical University
Syracuse, NY

[4]Department of Molecular Microbiology and Immunology
Department of Pediatrics
Brown University Medical School
Providence, RI 02918

*Corresponding author
Dr. Zhou Zhu,
Email: zhou_zhu@brown.edu

Materials and Methods

RNA isolation and mRNA analysis

Total cellular RNA from lungs was extracted using Trizol Reagent (Invitrogen, Carlsbad, CA) with a TissueLyser II (Qiagen, Valencia, CA). The mRNA of specific genes, CCL2 (MCP-1), CCL11 (Eotaxin-1), Arginase-1, Angiopoietin-1, IL-5 and IL-13, was evaluated by RT-PCR and quantitative RT-PCR with specific primers. For RT-PCR, amplified PCR products were analyzed by electrophoresis, and the intensity of the bands and the ratio of specific mRNA to β-Actin were analyzed with the Bio-Rad Gel Doc system and the Quantity One 4.4.1 software (Bio-Rad Laboratories, Hercules, CA). The ΔΔCt method was used for quantitative PCR. Reactions were carried out in an ABI 7900 real-time PCR system (Life Technologies, Carlsbad, CA) and values were expressed relative to house-keeping gene GAPDH. Primer sequences for RT-PCR and quantitative real-time PCR are shown in Table 1.

Results

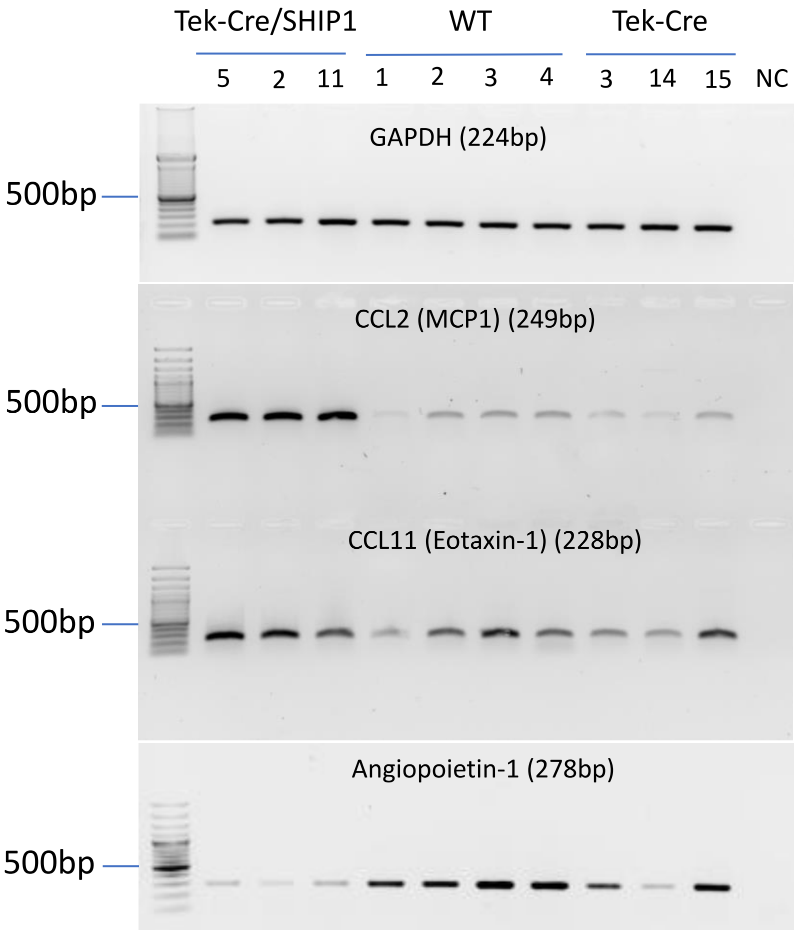

Figure S1A. RT-PCR analysis of gene expression in lung tissues of Tek-Cre/SHIP-1 and WT mice. Genes analyzed include: CCL2 (MCP-1), CCL11 (Eotaxin-1), and Angiopoietin-1. GAPDH gene was used as loading control. Left lane: DNA markers; Numbers: mouse number from different genotype groups; NC: Negative Control (no sample).

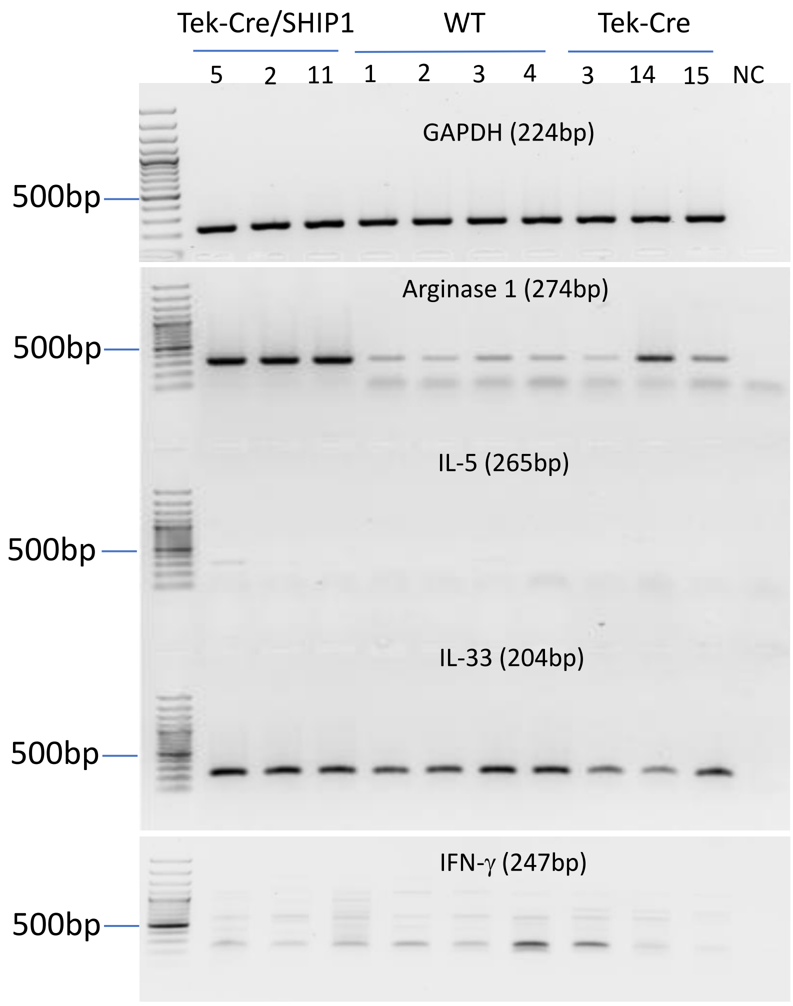

Figure S1B. RT-PCR analysis of gene expression in lung tissues of Tek-Cre/SHIP-1 and WT mice. Genes analyzed include: Arginase 1, IL-5*, IL-33, IFN-γ. GAPDH gene was used as loading control. Left lane: DNA markers; Numbers: mouse number from different genotype groups; NC: Negative Control (no sample).
